# Intra-erythrocytic vacuoles in asplenic patients: elusive genesis and original clearance of unique organelles

**DOI:** 10.3389/fphys.2023.1324463

**Published:** 2023-12-18

**Authors:** Lucie Dumas, Camille Roussel, Pierre Buffet

**Affiliations:** ^1^ Université Paris Cité and Université des Antilles, Inserm, Biologie Tissulaire du Globule Rouge, Paris, France; ^2^ Laboratoire d’Excellence GR-Ex, Paris, France; ^3^ Laboratoire d’Hématologie, Hôpital Universitaire Necker Enfants Malades, Assistance Publique–Hôpitaux de Paris (AP-HP), Paris, France; ^4^ Université Paris Cité, Service de Maladies Infectieuses et Tropicales Hôpital Universitaire Necker Enfants Malades, Assistance Publique–Hôpitaux de Paris (AP-HP), IHU Imagine, Paris, France

**Keywords:** spleen, hyposplenism, asplenia, red blood cell, pocked RBC, pitted RBC, vacuoles

## Abstract

The spleen plays a dual role of immune response and the filtration of red blood cells (RBC), the latter function being performed within the unique microcirculatory architecture of the red pulp. The red pulp filters and eliminates senescent and pathological RBC and can expell intra-erythrocytic rigid bodies through the so-called pitting mechanism. The loss of splenic function increases the risk of infections, thromboembolism, and hematological malignancies. However, current diagnostic tests such as quantification of Howell-Jolly Bodies and splenic scintigraphy lack sensitivity or are logistically demanding. Although not widely available in medical practice, the quantification of RBC containing vacuoles, i.e., pocked RBC, is a highly sensitive and specific marker for hyposplenism. The peripheral blood of hypo/asplenic individuals contains up to 80% RBC with vacuoles, whereas these pocked RBC account for less than 4% of RBC in healthy subjects. Despite their value as a spleen function test, intraerythrocytic vacuoles have received relatively limited attention so far, and little is known about their origin, content, and clearance. We provide an overview of the current knowledge regarding possible origins and mechanisms of elimination, as well as the potential function of these unique and original organelles observed in otherwise “empty” mature RBC. We highlight the need for further research on pocked RBC, particularly regarding their potential function and specific markers for easy counting and sorting, which are prerequisites for functional studies and wider application in medical practice.

## Introduction

**FIGURE 1:**
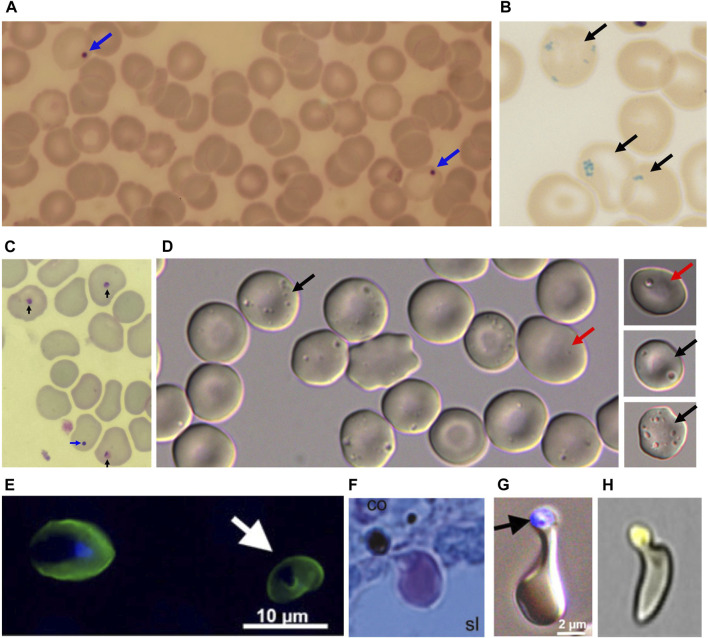
**(A)** Blue arrows: Howell Jolly Bodies (HJB) following Giemsa coloration. **(B)** Dark arrows: Pappenheimer bodies after Prussian blue coloration. **(C)** Blue arrow: HJB, Dark arrows: dead malaria parasites after Giemsa coloration. **(D)** pocked RBC with one vacuole (dark arrows) or multiple vacuoles (red arrows). **(E)** Left: Plasmodium falciparum-infected RBC by epifluorescence, visualized by anti-Resa antibody, staining the plasma membrane, and the DNA marker DAPI staining the intracellular parasite nucleus. Right (White arrow): Once-infected RBC stained by the anti-Resa antibody, from which the parasite has been expelled by pitting, hence the absence of DAPI staining. **(F)**. P. falciparum-infected RBC, pre-exposed to artesunate before perfusion ex-vivo in a human spleen, squeezing through an inter-endothelial slit with the dead parasite remnant retained upstream from the slit (Giemsa-stained histological section). **(G)** Same aspect generated by filtration through layers of microspheres (Epifluorescence and bright field, with dead parasite remnant stained with DAPI). **(H**) “Herniated” RBC from a patient with sickle cell disease. The cell trace yellow-stained vacuole looks almost expelled from the RBC. **(E and G)** from Ndour et al JID 2015; **(F)** from Buffet at al. Blood 2006; **(H)** from Sissoko et al. AJH 2022, with permission.

The spleen displays a dual immunologic and cell filtering function, and its parenchyma contains white pulp and red pulp. The marginal/perifollicular zone lies in between and likely connects these distinct functions. The white pulp and the marginal/perifollicular zone initiate the innate and adaptative immune responses. The latter traps and removes blood-borne antigens from the circulation. Based on a unique microvasculature architecture, the red pulp eliminates intact RBC (culling mechanism), ghosts, or small cellular components (pitting mechanism). Pitting is a spleen-specific process whereby intra-erythrocyte rigid bodies (i.e., Howell Jolly bodies, Pappenheimer bodies, dead malaria parasites, or intraerythrocytic vacuoles, Figure 1; Howell, 1890, Pappenheimer et al., 1945, Jauréguiberry et al., 2014, Box 1) are expelled from the RBC without lysis. These spleen functions can be partially or totally impaired, leading to hyposplenism or asplenia, respectively. The main cause of asplenia is splenectomy which is mainly performed for traumatic spleen injury, pancreatic cancer, hematologic malignancy, or inherited or auto-immune hematologic diseases. The main medical conditions that lead to a partial or complete loss of splenic function are sickle cell disease (SCD), celiac and inflammatory bowel diseases, and auto-immune disorders (William and Corazza, 2007; Corazza et al., 1982). The loss of splenic function is associated with an increased risk of infections with encapsulated bacteria (including the rare but very severe Overwhelming Post-Splenectomy Infection Syndrome) and a higher incidence of thromboembolism and cancer (Kristinsson et al., 2014). Reference methods to measure spleen filtering function are splenic scintigraphy and quantification of Howell Jolly Bodies (HJB). Scintigraphy requires the infusion of (mildly) radioactive material and is expensive. Results are semi-quantitative and few patients/guardians now accept this procedure. The quantification of HJB (that are DNA-containing intra-erythrocytic bodies) (Felka et al., 2007) is performed on venous blood and is also semi-quantitative. It lacks sensitivity, and is operator-dependent (Casper et al., 1976; Sills and Oski, 1979), although optimized quantification methods have recently emerged (El Hoss et al., 2018). Though not widely used, another specific and sensitive marker exists (Sills et al., 1988). This marker, conventionally called “pitted cells”, more accurately “pocked RBC” (Holroyde et al., 1969), corresponds to RBC containing vacuoles and has been observed for the first time by Koyama in 1962 (Koyama, 1962). Sissoko *et al.* compared specificity and sensitivity of pocked RBC and HJB. ROC curves in healthy and splenectomized subjects (Sissoko et al., 2022) showed that the most effective thresholds for the best diagnostic performance were 0.15% of RBC for HJB and 10% for pocked RBC. With these threshold, specificity and sensitivity of pocked RBC counts as a diagnostic test for hyposplenism were 100% for pocked RBC, and 96% and 91% for HJB, respectively (Sissoko et al., 2022). Patients with asplenia have impaired splenic filtration of RBC but also defective immune functions, as indicated by abnormal levels of lymphocytes subpopulations and tuftsin, a soluble marker. Tuftsin, that stimulates phagocytosis, displays decreased plasma levels in splenectomized patients compared to healthy subjects (Constantopoulos et al., 1973). Its activity is negatively correlated with the concentration of pocked RBC (Zoli et al., 1994). The concentration of memory B cells (switched and unswitched) is lower in splenectomized than in control subjects (Cameron et al., 2011; Lammers et al., 2012). This review is focused on RBC-related markers of hyposplenism.

BOX 1 **| Relevance of models to study pitting**

An *in vivo* spleen model relevant to human physiology would be helpful to study the mechanism of pitting and the genesis of vacuoles. Spleen anatomy and function differ between animal species, including *Homo sapiens*. Spleens of equine and feline are reservoirs of RBC unlike those of humans, rabbits, dogs and mice. Spleens of humans, rats and dogs display a sinusal structure as opposed to spleens of mice, cats and horses (Bowdler, 2001). In splenectomized mice, rats, and rabbits, vacuoles have been observed in RBC but their proportion increase only slightly, to less than 10% of all RBC after splenectomy (Buchanan et al., 1987; Shet et al., 2008). In mice and rats, the mean proportion of pocked RBC quantified by DIC was 0.4% and 3%, 63 days and 100 days post-splenectomy, respectively (Buchanan et al., 1987; Shet et al., 2008). In rabbits, a transient increase from 2.5% to 10% in the first 45 days was observed, but the proportion returned to baseline 90 days after splenectomy (Buchanan et al., 1987). Only dogs showed proportions similar to those observed in humans with pocked RBC reaching a plateau at 100 days post-splenectomy. However, the maximal rate of pocked RBC was lower than in humans (13%–39% at 100 days) (Buchanan et al., 1987). The most accurate observations are thus performed in human subjects and/or human tissues. *Ex-vivo* perfusion of human spleens preserves key RBC filtering functions, including pitting, but is a demanding, very low throughput model (Buffet et al., 2006).

Although more than half of circulating RBC in asplenic patient contain vacuoles, little is known about their genesis, content and clearance. We review here current knowledge about possible origins and mechanisms of elimination of these unique and original organelles, observed in otherwise “empty” mature RBC.

### Methods

Articles reviewed here were selected on google scholar using the following keywords: “pocked cells” OR “pitted cells” OR “pocked red blood cells” OR “pitted red blood cells” AND “sickle cell disease” OR “splenectomy” as of 21 July 2023. Additional articles not found by these keywords-based queries were selected from references of the selected articles, and personal collection of the authors.

### Pocked red blood cells counts to quantify hyposplenism

To quantify pocked RBC, whole blood is collected in tubes containing EDTA or heparin (de Haan et al., 1988; Reinhart and Chien, 1988). Pocked RBC are fixed in 0.1%–3% PBS-buffered glutaraldehyde (Pearson et al., 1979; Sills and Oski, 1979) or in 2% PBS-buffered paraformaldehyde (Nardo-Marino et al., 2022). Counts are against 250 to 2000 RBC under the oil-immersion objective (x1000) using differential interference contrast microscope (DIC Nomarski optics) (Pearson et al., 1979; Sills and Oski, 1979). Such fixed RBC can be stored for at least 1 month at room temperature, and at least 24 months at 4°C, after glutaraldehyde fixation (Holroyde and Gardner, 1970), and 6 weeks at 37°C or room temperature after paraformaldehyde fixation (Nardo-Marino et al., 2022). Recently, two automated quantification methods of pocked RBC were published: one using deep neural network analysis (Nardo-Marino et al., 2022), the other imaging flow cytometry after fluorescent labeling (Sissoko et al., 2022). Marino *et al.* generated a learning model from DIC pictures (Nardo-Marino et al., 2022). This automated count was reliable and correlated with manual counts (albeit with lower performance when the proportion of pocked RBC was high). Sissoko *et al.* observed the presence of spotted RBC after labeling with Cell Trace Yellow. The proportion of these spotted RBC correlated with the proportion of pocked RBC counted manually by DIC (Sissoko et al., 2022).

In healthy subjects, pocked RBC generally account for less than 4% of RBC (Holroyde et al., 1969; Holroyde and Gardner, 1970; Casper et al., 1976) while in splenectomized patients, the proportion of pocked RBC varies between 20% and 80% (Koyama, 1962; Holroyde and Gardner, 1970). There is a good correlation between splenic scintigraphy, HJB, and pocked RBC (Rogers et al., 2011; El Hoss et al., 2019) but HJB are generally not observed when pocked RBC counts are lower than 8% (Phoon, 1997). The marked difference in proportions of pocked RBC between controls and hyposplenic subjects makes it a robust quantitative method (Phoon, 1997). Phoon *et al.* defined hyposplenism by the presence of 3.5%–10% pocked RBC, and asplenia by the presence of more than 12% of pocked RBC (Phoon, 1997). However, none of the thresholds published so far have been validated by large-scale clinical studies correlating them with the risk of long-term complications. Of note, this marker is irrelevant in neonates where 5%–40% of RBC are pocked (Holroyde et al., 1969; Kim et al., 1980). This high rate is probably associated with spleen developmental immaturity. The proportion of pocked RBC returns to normal (low) levels during the first month of life.

### Vacuoles are morphologically diverse

Vacuoles in pocked RBC appear by DIC microscopy as punctate light or dark craters (Figure 1) (Koyama, 1962). Pocked RBC contain single or multiple vacuoles in hyposplenic subjects (0.2µm–1.5 µm diameter) and only a single and generally small vacuole in healthy subjects (0.2µm–0.8 µm diameter) (Holroyde and Gardner, 1970; Sills and Oski, 1979). By transmission electron microscopy (TEM), Reinhart *et al.* observed that 13% of RBC pictures contained small vacuoles (40 and 330 nm) in healthy subject, either empty (1.7% of RBC) or containing small grainy material (11.3% of RBC) (Reinhart and Chien, 1988). In the same study, RBC pictures with small vacuoles were four times more frequent in splenectomized patients than in healthy individuals (42.5% of RBC pictures) and were empty (36.2% of RBC pictures) or containing dense material (6.3% of RBC pictures). In these patients, the authors also observed low proportions of RBC with large vacuoles (diameter >300 nm) and clusters of small vacuoles (3.2% and 2.6% respectively). Kent *et al.* described by TEM larger vacuoles (200–1000 nm), containing electron dense material, in healthy subjects as well as in patients with anemia, with or without splenectomy (Kent et al., 1966). Large vacuoles seen optically may correspond to the coalescence of small vacuoles seen by TEM, which may ultimately fuse to generate a single large vacuole (Holroyde and Gardner, 1970; Reinhart and Chien, 1988). When vacuoles content was studied by TEM (Kent et al., 1966; Holroyde and Gardner, 1970; Schnitzer et al., 1971), they appeared either empty or heterogeneous, and sometimes contained material identified as altered organelles like mitochondria, ribosomes and smooth internal membranes surrounded by an external membrane (Kent et al., 1966; Holroyde and Gardner, 1970; Schnitzer et al., 1971). These observations were based exclusively on morphological characteristics and, so far, no immunostaining has confirmed that these structures are altered organelles. Other vacuoles have a homogenous internal structure, similar to the cytoplasm in aspect and density, which suggests that they may contain hemoglobin (Kent et al., 1966; Schnitzer et al., 1971). Another subtype of iron-containing vacuoles has been observed, that may correspond to Pappenheimer bodies (Kent et al., 1966). Finally, few autophagic vacuoles with a phosphatase acid activity have been described (Kent et al., 1966). These observations do not robustly establish the origin of vacuoles and their content.

This wealth of morphological observations leaves indeed significant knowledge gaps (Please see Box 2). There is currently no method to sort pocked RBC or to isolate vacuoles. Therefore, it remains uncertain whether all TEM observations of vacuoles correspond to those observed by DIC. As mentioned above, several subtypes of vacuoles have been observed by TEM, suggesting that vacuoles may have multiple origins. Small (<400 nm) vacuoles observed by TEM cannot be seen by DIC and have been observed in RBC from healthy subject in which the proportion of pocked RBC is usually very low. Largest vacuoles containing structures identified as altered organelles may correspond to remnants of reticulocyte multivesicular bodies but, as mentioned, no direct staining has confirmed this hypothesis so far.

BOX 2 **| Knowledge gaps and current research questions**

1. The fine mechanism of pitting, responsible for vacuole clearance, is only partially understood. It may involve inter-endothelial slits, splenic macrophages, or a synergistic contribution of both.
2. The origin and composition of small vacuoles present in almost all RBC is unknown.
3. The origin and composition of large, complex vacuoles, containing organelle-like remnants is unknown.
4. How the coalescence of small vacuoles generates larger, optically observable, vacuoles is not known.
5. The role of these vacuoles in the RBC physiology is unknown.
6. The theranostic value of pocked RBC is not robustly established.

### Genesis of vacuoles: confronting hypotheses

Following splenectomy, a gradual increase in the proportion of pocked RBC is observed, and the plateau is reached after 60–100 days (Koyama, 1962; Zago et al., 1986; Buchanan et al., 1987), or 130 days in one study (de Haan et al., 1988). Vacuoles may appear during erythropoiesis and persist as remnants in mature RBC. Alternatively, they may be created *de novo* in circulating RBC (Reinhart and Chien, 1988). To solve the quandary, searchers have quantified vacuoles in young *versus* old RBC (de Haan et al., 1988; Reinhart and Chien, 1988). Young and old RBC were separated based on density using microhematocrit tubes (Reinhart and Chien, 1988), or angle-head centrifugation (de Haan et al., 1988). Using DIC microscopy on RBC from splenectomized subjects, Reinhart *et al.* found 2.6 times more pocked RBC in old than in young cells (52.2% vs 20.8%). De Haan *et al.* reported a similar difference (42.2% vs 27.6%) as well as a continuous increase of pocked RBC both in young and old RBC between 10 and 130 days after splenectomy (de Haan et al., 1988). A positive correlation between the proportion of pocked RBC and levels of glycated hemoglobin (HbA1c), a marker of RBC aging, was also observed (de Haan et al., 1988). Selective case reports also support the hypothesis that most vacuoles appear *de novo* in mature RBC. In two patients with idiopathic autoimmune hemolytic anemia unresponsive to splenectomy (that had been performed 17 and 54 months previously), Zago *et al.* observed that therapy with corticosteroids improved RBC survival and reduced reticulocytosis. In parallel, 12 weeks after starting treatment, the proportion of pocked RBC had increased from 5.5% to 29.8% in one patient; and from 13% to 47.4% in the other (Holroyde and Gardner, 1970). Similarly, when a splenectomized patient was transfused with normal RBC for pure red cell aplasia, Holroyde *et al.* observed a dilution of pocked RBC immediately after transfusion (from 44% to 26% of RBC) followed by a rapid increase and return to high baseline proportions in about 20 days, suggesting that vacuoles had appeared in transfused RBC, which were most likely mature in vast majority (Holroyde and Gardner, 1970).

A theoretical argument against the hypothesis of *de novo* appearance of vacuoles is the elusive mechanism of their (potential) creation in mature RBC, a highly differentiated enucleated cell entity devoid of organelles and endocytosis machinery. The formation of intracellular vacuoles is a well-known mechanism in erythroid precursors but the mechanism underlying their suspected appearance in mature RBC remains currently an enigma. Several teams have tried to replicate experimentally this process *in vitro* (Reinhart and Chien, 1988; Sills et al., 1988; Colin and Schrier, 1991). Incubating RBC with 0.5 mM chlorpromazine for 2 min at 37°C induced the appearance of vacuoles similar in shape and size (40–234 nm) to that observed in healthy subjects, as observed by TEM (Reinhart and Chien, 1988). In a more physiological approach, Sills *et al.* compared the creation of vacuoles *in vitro* (upon incubation of normal RBC for 6 days at 37°C in physiologic buffer or plasma), to the *in vivo* formation of vacuoles during the same period immediately after splenectomy (Sills et al., 1988). The proportion of vacuoles increased at a similar pace in these two groups: from 0.3% to 4.9% *in vitro* and 0.4%–4.1% in patients, suggesting that vacuoles do appear in mature RBC. The experience *in vitro* could not be extended further because of the increasing crenation of RBC (echinocytosis) when they were incubated for more than a week. Crenation impairs an accurate visualization of vacuoles. Colin *et al.* performed the same experiment with and without FITC-BSA in order to determine whether vacuoles could be created by the invaginations of the plasma membrane (Colin and Schrier, 1991). No fluorescence was observed inside RBC, despite the appearance of vacuoles after 144 h of incubation in medium containing FITC-BSA. Conversely, in the presence of the antimalarial drug primaquine, the appearance of vacuoles was accompanied by intra-erythrocytic fluorescence, which strongly suggested that these primaquine-induced vacuoles, unlike “spontaneous” vacuoles, were created by internalization of the plasma membrane (Colin and Schrier, 1991). How vacuoles spontaneously appear *in vitro* and *in vivo* is therefore still mysterious. Spontaneous appearance of vacuoles in Eagle’s modified buffer was not inhibited by the addition of sodium vanadate (that inhibits ATPase activity required for drug-induced endocytosis), NaF (that inhibits ATP production by glycolysis thereby blocking endocytosis), or NaCN (which inhibits transferrin receptor-mediated endocytosis). The process is therefore ATP- and transferrin receptor-independent, and, in the absence of primaquine (to which splenectomized patients are generally not exposed), is probably not due to plasma membrane invagination (Colin and Schrier, 1991). If the experimental appearance of intra-erythrocytic vacuoles *in vitro* replicates the natural mechanism in action in hypo- or a-splenic patients, they are not generated by an endocytosis-like mechanism. One hypothesis that reconciles all currently available information would be the progressive coalescence (both *in vitro* or *in vivo*) of preexisting smaller vacuoles created during erythropoiesis (Reinhart and Chien, 1988). They would “appear” optically in mature RBC but from elements already present, too small to be seen by DIC, but observable by TEM.

As a further source of complexity, vacuoles are morphologically diverse. The different types may therefore stem from different mechanisms. Empty vacuoles and those containing homogenous material resembling RBC cytoplasm may be “created” (probably by coalescence, see above) *de novo* in circulation, and would be removed rapidly by the spleen, except in subjects with hyposplenism. Larger and more complex vacuoles containing organelle-like components may be remnants from erythropoiesis never removed from the mature RBC in asplenic subjects. Indeed, no known autonomous, active process enabling their expulsion persists in mature RBC. As summarized at the previous section, the appearance of optically visible vacuoles in mature RBC is supported by several observations (Holroyde and Gardner, 1970; de Haan et al., 1988; Reinhart and Chien, 1988). That the minority of “complex” vacuoles are remnants from erythropoiesis is a logical assumption but leans only on TEM pictures (Kent et al., 1966; Holroyde and Gardner, 1970; Schnitzer et al., 1971). Currently, there is no way to differentiate these two types of vacuoles by other methods.

### Clearance of intra-erythrocytic vacuoles

The observation that high proportions of pocked RBC are present only in splenectomized subjects strongly suggests that vacuoles are eliminated by the spleen (Koyama, 1962; Kent et al., 1966). The demonstration was made in humans by Holroyde *et al.* who transfused a normal subject with ^51^Cr labelled RBC from splenectomized donor having 49.5% of pocked RBC (Holroyde and Gardner, 1970). Seventy hours after transfusion, 90% of ^51^Cr RBC were still in circulation in the recipient, while the proportion of pocked RBC had dropped to 0 (Holroyde and Gardner, 1970). Buchanan *et al.* performed the same experiment in dogs: pocked RBC counts decreased from 6.5% to 1% in 170 h after transfusion while transfused RBC were still in circulation (Buchanan et al., 1987). This persistence of labeled RBC with disappearance of pocked RBC is strongly reminiscent of a prior observation by Crosby using RBC containing Pappenheimer bodies (Crosby and Benjamin, 1957) that forged the concept of pitting. Pitting was later observed with malaria parasites, with initial suggestive pictures by TEM (Schnitzer et al., 1972) later confirmed by RBC labeling (Angus et al., 1997; Chotivanich et al., 2002). Searchers in Thailand observed a rapid parasite clearance (in less than a week) in spleen-intact malaria patients treated with artemisinins, followed by the appearance in circulation of uninfected RBC labeled with a parasitic protein, called RESA (Ring-Erythrocyte Surface Antigen) (Angus et al., 1997; Chotivanich et al., 2002). This peculiar subpopulation was called “once-infected RBC”. This process was later replicated in human spleens perfused *ex-vivo* (Buffet et al., 2006) enabling the visualization on histological sections of infected RBC squeezing through narrow splenic slits with the dead parasite remnant laying upstream from the slit (Figure 1). In splenectomized subjects, post-treatment parasite clearance is much longer, often lasting several weeks, without appearance of “once infected” RESA-positive RBC in circulation (Angus et al., 1997; Chotivanich et al., 2002). This suggests that vacuoles are eliminated from RBC by pitting, enabling the almost intact pitted RBC to go back into circulation. A similar process is likely operating for Howell-Jolly bodies and Pappenheimer bodies. Pitting likely requires squeezing through inter-endothelial slits of the spleen, selective engulfment of the vacuole by macrophages, or the synergistic contribution of both mechanisms. Ndour *et al.* performed microsphiltration experiments, where layers of microspheres of different sizes mimic inter-endothelial slits (Ndour et al., 2015). Using RBC infected by the malaria parasite *Plasmodium falciparum* and exposed to artesunate, they quantified “once-infected” RESA-positive RBC, before and after microsphiltration. A low (<5%) rate of pitting was observed, lower than the rate observed *in vivo*, generally greater than 50% (Ndour et al., 2015). Anyona *et al.* co-incubated infected RBC with THP1 monocytes and observed the appearance of uninfected, RESA-positive RBC (Anyona et al., 2006). Pocked RBC are also pitted by the spleen and a similar uncertainty exists regarding the specific cellular mechanism leading to this expulsion of vacuoles (Nagelkerke et al., 2018).

### Discussion

**FIGURE 2:**
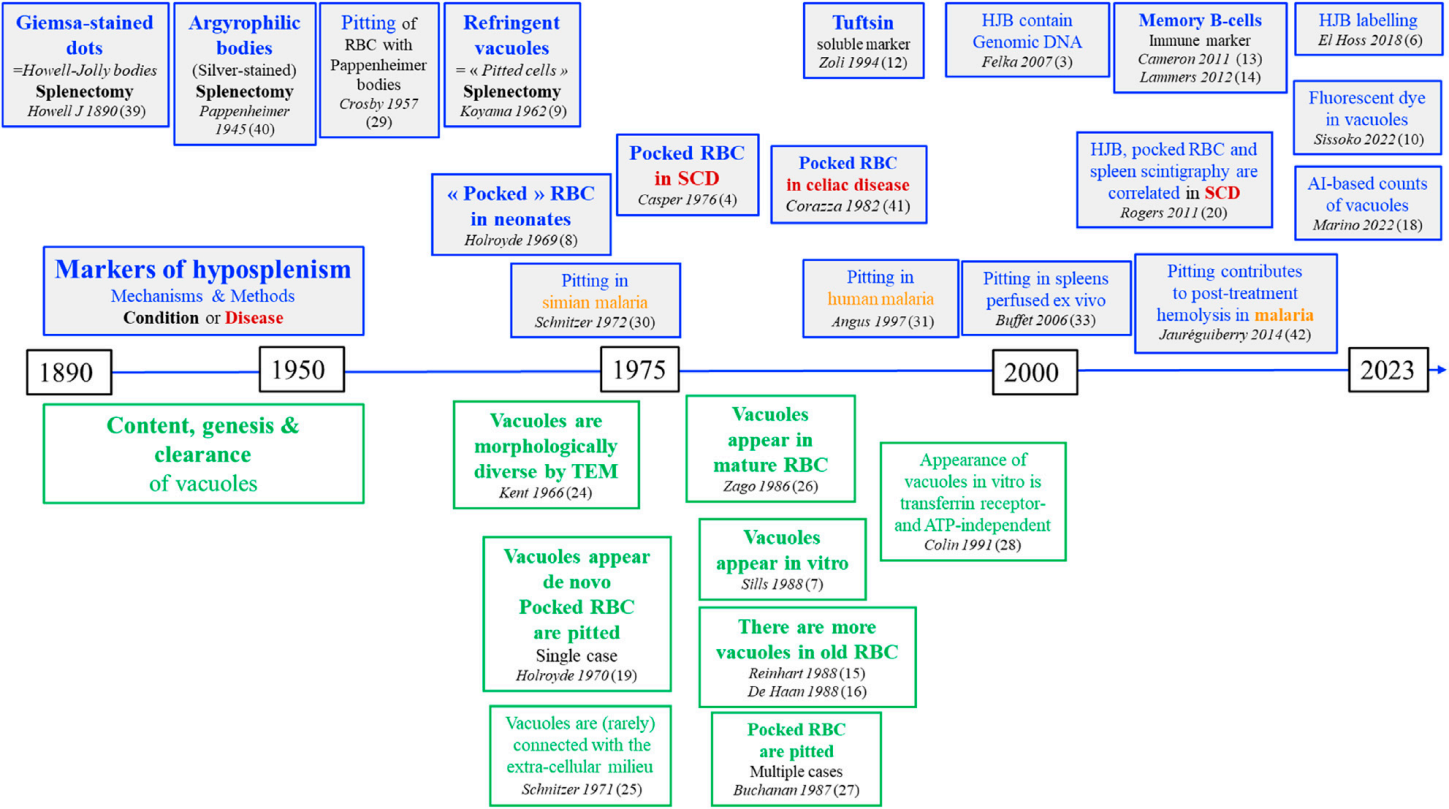
Timeline of the main discoveries on markers of splenic function.

Pocked RBC were first reported in 1962 and have since received relatively limited attention. Large studies to validate their quantitative theranostic value are still missing. Their clearance, or, more accurately said, the clearance of their vacuoles by pitting is better understood than their genesis (Figure 2). Several teams have shown that vacuoles, rather than being former intra-erythroblastic organelles, may appear in circulating mature RBC, likely by a process distinct from conventional endocytosis. Yet, by TEM, residues resembling organelles are observed in a proportion of vacuoles. “Empty” vacuoles, which are the majority, likely appear by coalescence in mature RBC, whereas the minority of more complex and large vacuoles may be remnants from erythropoiesis. Not least, the possible function of intra-erythrocytic vacuoles (if there is any) has been poorly explored so far. Small vacuoles (visible only by TEM) are present in almost all RBC, including those from healthy subjects, which suggests they may have a (yet to be identified) physiological role. Because of their high numbers in asplenic individual, a pathogenic (e.g., procoagulant) role is also conceivable. A better knowledge of the composition of each subtype of vacuoles would help identify specific markers, for simple counting and sorting, as prerequisite for functional studies and wider use in medical practice.
